# Extracellular HMGB1 exacerbates autoimmune progression and recurrence of type 1 diabetes by impairing regulatory T cell stability

**DOI:** 10.1007/s00125-020-05105-8

**Published:** 2020-02-19

**Authors:** Jing Zhang, Longmin Chen, Faxi Wang, Yuan Zou, Jingyi Li, Jiahui Luo, Faheem Khan, Fei Sun, Yang Li, Jing Liu, Zhishui Chen, Shu Zhang, Fei Xiong, Qilin Yu, Jinxiu Li, Kun Huang, Bao-Ling Adam, Zhiguang Zhou, Decio L. Eizirik, Ping Yang, Cong-Yi Wang

**Affiliations:** 1 The Center for Biomedical Research, Tongji Hospital Research Building, Tongji Hospital, Tongji Medical College, Huazhong University of Science and Technology, Wuhan, 430000 Caidian China; 2 Department of Respiratory and Critical Care Medicine, NHC Key Laboratory of Respiratory Disease, Tongji Hospital, Tongji Medical College, Huazhong University of Science and Technology, Wuhan, China; 3 Key Laboratory of Organ Transplantation, Ministry of Education, NHC Key Laboratory of Organ Transplantation, Key Laboratory of Organ Transplantation, Chinese Academy of Medical Sciences, Tongji Hospital, Wuhan, China; 4 Shenzhen Third People’s Hospital, Shenzhen, Guangdong China; 5 Tongji School of Pharmacy, Tongji Medical College, Huazhong University of Science and Technology, Wuhan, China; 6 Department of Surgery, Medical College of Georgia at Augusta University, Augusta, GA USA; 7 Diabetes Center, The Second Xiangya Hospital, Institute of Metabolism and Endocrinology, Central South University, Changsha, China; 8 ULB Center for Diabetes Research, Université Libre de Bruxelles, Brussels, Belgium

**Keywords:** Beta cell mass turnover, Diabetes reversal, High-mobility group box 1, HMGB1, Islet transplantation, Regulatory T cells, Type 1 diabetes

## Abstract

**Aims/hypothesis:**

High-mobility group box 1 (HMGB1), an evolutionarily conserved chromosomal protein, was rediscovered to be a ‘danger signal’ (alarmin) that alerts the immune system once released extracellularly. Therefore, it has been recognised contributing to the pathogenesis of autoimmune diabetes, but its exact impact on the initiation and progression of type 1 diabetes, as well as the related molecular mechanisms, are yet to be fully characterised.

**Methods:**

In the current report, we employed NOD mice as a model to dissect the impact of blocking HMGB1 on the prevention, treatment and reversal of type 1 diabetes. To study the mechanism involved, we extensively examined the characteristics of regulatory T cells (Tregs) and their related signalling pathways upon HMGB1 stimulation. Furthermore, we investigated the relevance of our data to human autoimmune diabetes.

**Results:**

Neutralising HMGB1 both delayed diabetes onset and, of particular relevance, reversed diabetes in 13 out of 20 new-onset diabetic NOD mice. Consistently, blockade of HMGB1 prevented islet isografts from autoimmune attack in diabetic NOD mice. Using transgenic reporter mice that carry a *Foxp3* lineage reporter construct, we found that administration of HMGB1 impairs Treg stability and function. Mechanistic studies revealed that HMGB1 activates receptor for AGE (RAGE) and toll-like receptor (TLR)4 to enhance phosphatidylinositol 3-kinase (PI3K)–Akt–mechanistic target of rapamycin (mTOR) signalling, thereby impairing Treg stability and functionality. Indeed, high circulating levels of HMGB1 in human participants with type 1 diabetes contribute to Treg instability, suggesting that blockade of HMGB1 could be an effective therapy against type 1 diabetes in clinical settings.

**Conclusions/interpretation:**

The present data support the possibility that HMGB1 could be a viable therapeutic target to prevent the initiation, progression and recurrence of autoimmunity in the setting of type 1 diabetes.

**Electronic supplementary material:**

The online version of this article (10.1007/s00125-020-05105-8) contains peer reviewed but unedited supplementary material, which is available to authorised users.

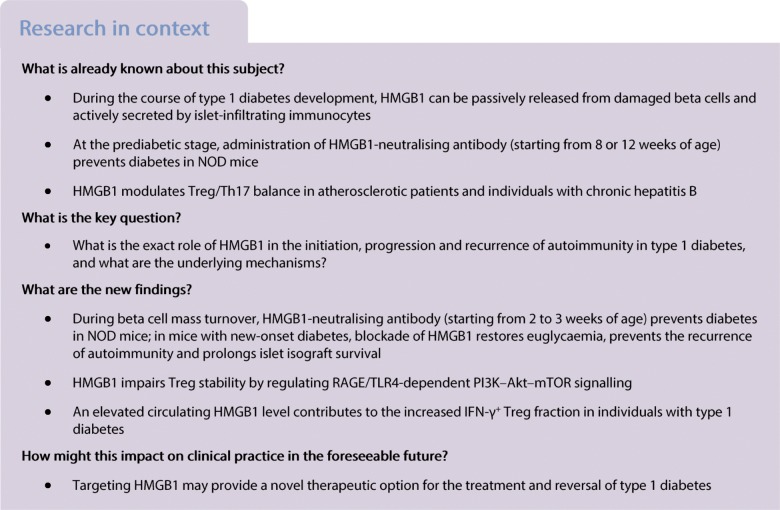



## Introduction

Type 1 diabetes is a chronic autoimmune disease resulting from a severe loss of the insulin-producing beta cells by autoantigens [1]. The pathological hallmark of type 1 diabetes is insulitis, an inflammatory lesion of the islet accompanied by beta cell loss. Inflammatory cells are observed in the islet periphery (peri-insulitis) or within the islet parenchyma [2], leading to a dialogue with the pancreatic beta cells that culminates in the destruction of beta cells [3]. The autoimmune destruction of pancreatic beta cells can precede sub-clinically over a long period of time, and overt diabetes develops only when the beta cell functional mass drops below the critical level needed to preserve normoglycaemia [4]. Insulin is usually administered to regulate blood glucose, but exogenously provided insulin cannot regulate blood glucose as accurately as endogenous insulin. Islet transplantation has been regarded as a promising alternative therapeutic approach, but the need for continuous immune suppression, plus the fact that most patients experience a progressive loss of islet graft function due to autoimmune/allogeneic immune rejection, metabolic stress and the cytotoxic effect of immunosuppressive regimens on beta cells, has significantly dampened enthusiasm for this approach [5–8].

High-mobility group box 1 (HMGB1) is one of the most evolutionarily conserved proteins in the eukaryotic kingdom. It is likely to have originated more than 500 million years ago before the split between the animal and plant kingdoms. As a nuclear protein, HMGB1 is associated with the regulation of nucleosomal structure and stability as well as the binding of transcription factors to their cognate DNA sequences [9–12]. In the late 1990s, HMGB1 was rediscovered as a late mediator of endotoxin lethality in murine models [13], and follow-up studies further revealed that HMGB1 can act as an innate ‘danger signal’ (alarmin) implicated in host defence and tissue repair. Our previous studies demonstrated that HMGB1 can be either passively released from damaged pancreatic beta cells or actively secreted by islet-infiltrating immunocytes such as dendritic cells (DCs) and macrophages [14, 15], and that blockade of HMGB1 in 8- or 12-week-old NOD mice delayed diabetes onset [15]. In the current study, we want to determine the prophylactic and therapeutic efficacy of HMGB1-neutralising antibody at different stages of type 1 diabetes, including reversal of disease and protection of islet isografts from recurrent autoimmune attack, and dissect the underlying mechanisms.

## Methods

### Animals

NOD/ShiLtJ mice were purchased from the Model Animal Research Center of Nanjing University (Nanjing, China). BALB/c and NOD-*scid* mice were purchased from Beijing HFK Bioscience (Beijing, China). 008694-NOD/ShiLt-Tg(*Foxp3*-EGFP/cre)1cJbs/J mice and 007914-B6.Cg-*Gt(ROSA)26Sor*^*tm14(CAG-tdTomato)Hze*^/J mice were purchased from the Jackson Laboratory (Bar Harbor, ME, USA). All mice were housed in a specific pathogen-free animal facility at the Tongji Medical College on a 12/12 h light/dark cycle. After the mice reached 12 weeks of age, female NOD mice were monitored for blood glucose three times per week using an Accu-Check Advantage glucometer (Roche Diagnostics, Indianapolis, IN, USA) and classed as diabetic once two consecutive blood glucose readings were >13.8 mmol/l. All protocols for animal studies were approved by the Tongji Hospital Animal Care and Use Committee in accordance with the National Institutes of Health guidelines.

### Production and purification of an HMGB1 neutralising antibody

We obtained an HMGB1-neutralising antibody using conventional hybridoma technology (See electronic supplementary material [ESM] Methods). The purity, specificity and titres against HMGB1 of the antibody were further confirmed (ESM Results and ESM Fig. 1).

### Diabetes reversal

Once mice became diabetic, serum was collected before treatment randomisation to measure insulin autoantibodies (IAAs) and HMGB1 levels (see ESM Methods). Next, the mice were randomly treated with either anti-HMGB1 (500 μg/mouse) or the same amount of normal mouse IgG (SLM56-0500; Equitech-Bio, Kerrville, TX, USA) as a control every other day for 2 weeks. One week after treatment, some of the mice were euthanised to collect blood and tissues. Cytokine levels, including TNF-α, IFN-γ, IL-1β, IL-4, IL-17A, TGF-β and IL-10, were determined using ELISA kits from BD Biosciences and eBioscience (both San Diego, CA, USA). Pancreases were subjected to H&E staining, immunohistochemical staining for HMGB1 (ab18256; Abcam, Cambridge, MA, USA), CD4 (25229s; Cell Signaling Technology, Danvers, MA, USA) and CD8 (98941s; Cell Signaling Technology, Danvers, MA, USA), and immunofluorescence staining for insulin (4590s; Cell Signaling Technology, Danvers, MA, USA) and glucagon (sc-130624; Santa Cruz Biotechnology, Santa Cruz, CA, USA). Insulitis was scored on the basis of islet infiltration by two pathologists in a blinded fashion. T cell subsets in the spleen, pancreatic lymph nodes (PLNs) and pancreas were analysed by flow cytometry (see ESM Methods for details).

### Islet transplantation

Pancreatic islets were isolated using established methods [16]. A total of 500 islets obtained from 4- to 5-week-old NOD donors were transplanted into diabetic female NOD mice under the left kidney capsule. Blood glucose was measured daily after transplantation. Once normoglycaemia was achieved, diabetes recurrence was defined as the first of 2 consecutive days of nonfasting blood glucose >13.8 mmol/l. Graft survival was calculated as the number of days before diabetes recurrence.

### Cell purification and culture

Naive T cells were enriched with a Naive CD4^+^ T Cell Isolation Kit for mouse (130-104-453; Miltenyi Biotec, Auburn, CA, USA), and regulatory T cells (Tregs) and conventional T cells (Tconv) were enriched with a CD4^+^CD25^+^ Regulatory T Cell Isolation Kit for mouse (130-091-041; Miltenyi Biotec) according to the manufacturer’s instructions. The sorted T cells were cultured in RPMI 1640 medium (plus β-mercaptoethanol) supplemented with 10% FBS, 1% GlutaMax, 1% sodium pyruvate, and 1% Pen/Strep (all from Gibco, Shanghai, China) for further study, as detailed in the ESM Methods.

### Methylation analysis of Treg cell-specific demethylated region

Methylation analysis of the *Foxp3* locus was performed as previously reported [17, 18]. See ESM Methods for details of analysis of Treg cell-specific demethylated region (TSDR).

### Real-time PCR and western blot analysis

Real-time PCR and western blot analysis were performed as previously reported [19]. Primer sequences for all examined genes are listed in ESM Table 1, and detailed information is described in the ESM Methods. Samples were excluded from analyses if mRNA or protein was not detected.

### In vitro suppression assays and T cell-transfer model of colitis

In vitro suppression assays and T cell-transfer model of colitis were conducted using established techniques [20, 21]. A score from 0 to 4 for intestinal lesions based on the number of lesions as well as their severity was applied in a blinded fashion by two examiners, and detailed information is available in the ESM Methods.

### Human samples

Blood samples were obtained from participants with type 1 diabetes and healthy control participants, and all of the study participants provided informed consent. All studies in humans were conducted in accordance with the NIH guidelines and were approved by the Institutional Review Board (IRB) of Tongji Hospital (TJ-IRB20160602).

### Statistical analysis

The Kaplan–Meier method was used for survival analysis. The logrank (Mantel–Cox) test was used to determine differences in diabetes incidence between the groups. The difference in insulitis severity was determined at each time point using the χ^2^ test. Other results were expressed as mean ± SEM, and their comparisons were accomplished by Student’s *t* test with 95% CI. All in vitro studies were conducted at least three times. In all cases, *p* < 0.05 was considered statistically significant. Statistical analyses of the data were conducted using GraphPad Prism 5 software (GraphPad Software, San Diego, CA, USA).

## Results

### Blockade of HMGB1 during beta cell mass turnover prevents diabetes in NOD mice

**Fig. 1:**
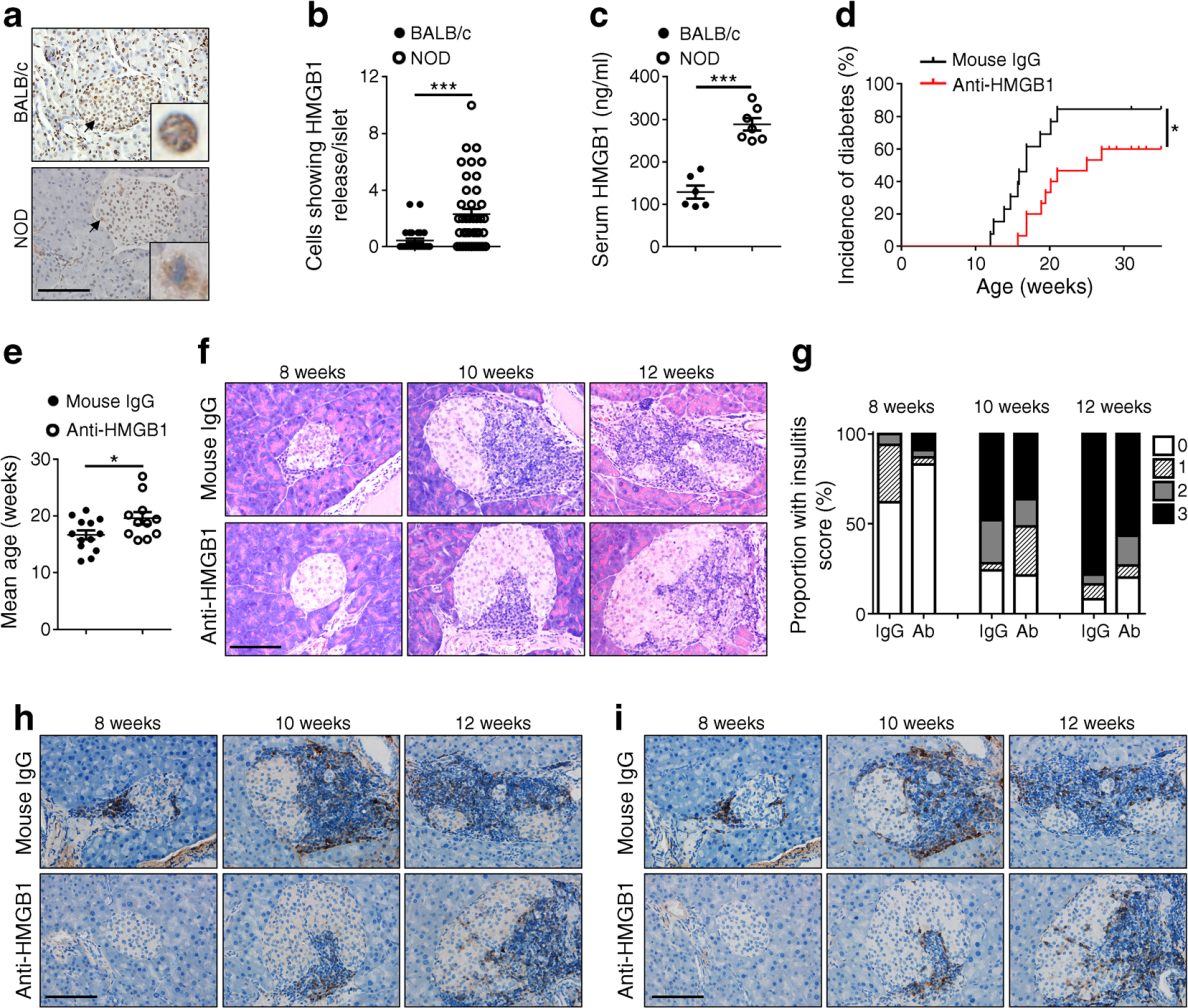
Blockade of extracellular HMGB1 during beta cell mass turnover prevents insulitis progression and diabetes onset in NOD mice. (**a**) Immunohistochemical staining of HMGB1 in pancreatic sections from BALB/c and NOD mice (representative of *n* = 6 for BALB/c mice and *n* = 7 for NOD mice, 2.5 weeks of age). Location of boxed magnifications is indicated by arrows. (**b**) Quantification of cells showing HMGB1 release per islet. (**c**) Analysis of serum HMGB1 levels in 2.5-week-old BALB/c (*n* = 6) and NOD (*n* = 7) mice. (**d**) The incidence of diabetes in mice treated with mouse IgG (*n* = 13) or anti-HMGB1 (*n* = 15), starting at 2.5 weeks of age. (**e**) The mean age of diabetes onset. (**f**) Representative results showing H&E staining of pancreatic sections. (**g**) A bar graph showing insulitis severity examined at 8, 10 and 12 weeks of age. Insulitis was scored as described in the Methods. (**h**, **i**) Representative results of immunohistochemical staining for CD4 (**h**) and CD8 (**i**) (brown staining) in sections of the pancreas. *n* = 4 per group at each time point (**f**–**i**). Scale bars, 100 μm (**a**, **f**, **h**, **i**); original magnification ×400 (**a**, **f**, **h**, **i**). Values are expressed as mean ± SEM. Statistical difference in (**d**) was analysed by a logrank test; in (**g**) was determined at 8-week (*p* < 0.001), 10-week (*p* < 0.001), and 12-week (*p* = 0.002) time points using the χ^2^ test; and in other figure parts was analysed by unpaired Student’s *t* test; *p < 0.05, ***p < 0.001. IgG, mouse IgG; Ab, anti-HMGB1

In general, mice exhibit beta cell apoptosis during beta cell mass turnover around 2 to 3 weeks of age, while NOD mice manifest secondary necrosis due to the defective clearance of apoptotic beta cells [14, 22–24]. Given that secondary necrosis is accompanied by the HMGB1 passive release [15], in situ HMGB1 immunohistochemical staining was conducted in pancreatic sections of 2.5-week-old NOD mice. Unlike 2.5-week-old BALB/c mice, in which HMGB1 was solely localised in the nuclei of islet cells, some islet cells in the NOD mice showed condensed nuclei and positive cytoplasmic HMGB1 staining (Fig. 1a,b), indicating that HMGB1 was passively released from secondary necrotic beta cells. In line with this observation, NOD mice manifested significantly higher levels of HMGB1 in the periphery than that in BALB/c mice during the course of beta cell mass turnover (Fig. 1c). We then treated 2.5-week-old NOD female mice with either an HMGB1-neutralising antibody (500 μg/mouse) or the same amount of normal mouse IgG every other day for two weeks. As expected, blockade of HMGB1 remarkably reduced diabetes incidence (84.6% vs 60%) (Fig. 1d) and delayed diabetes onset (16.7 ± 0.8 vs 19.6 ± 1.1 weeks) (Fig. 1e). Consistently, the severity of insulitis at each time point (8, 10 and 12 weeks of age) examined in anti-HMGB1-treated mice was significantly lower than that of IgG-treated mice (Fig. 1f,g). Decreased islet infiltration was further confirmed by CD4 (Fig. 1h) and CD8 (Fig. 1i) staining. Collectively, these data demonstrate that blockade of HMGB1 during early beta cell mass turnover prevented insulitis progression and, as a result, decreased the incidence of diabetes in NOD mice.

### Blockade of HMGB1 restores euglycaemia in mice with new-onset diabetes

**Fig. 2:**
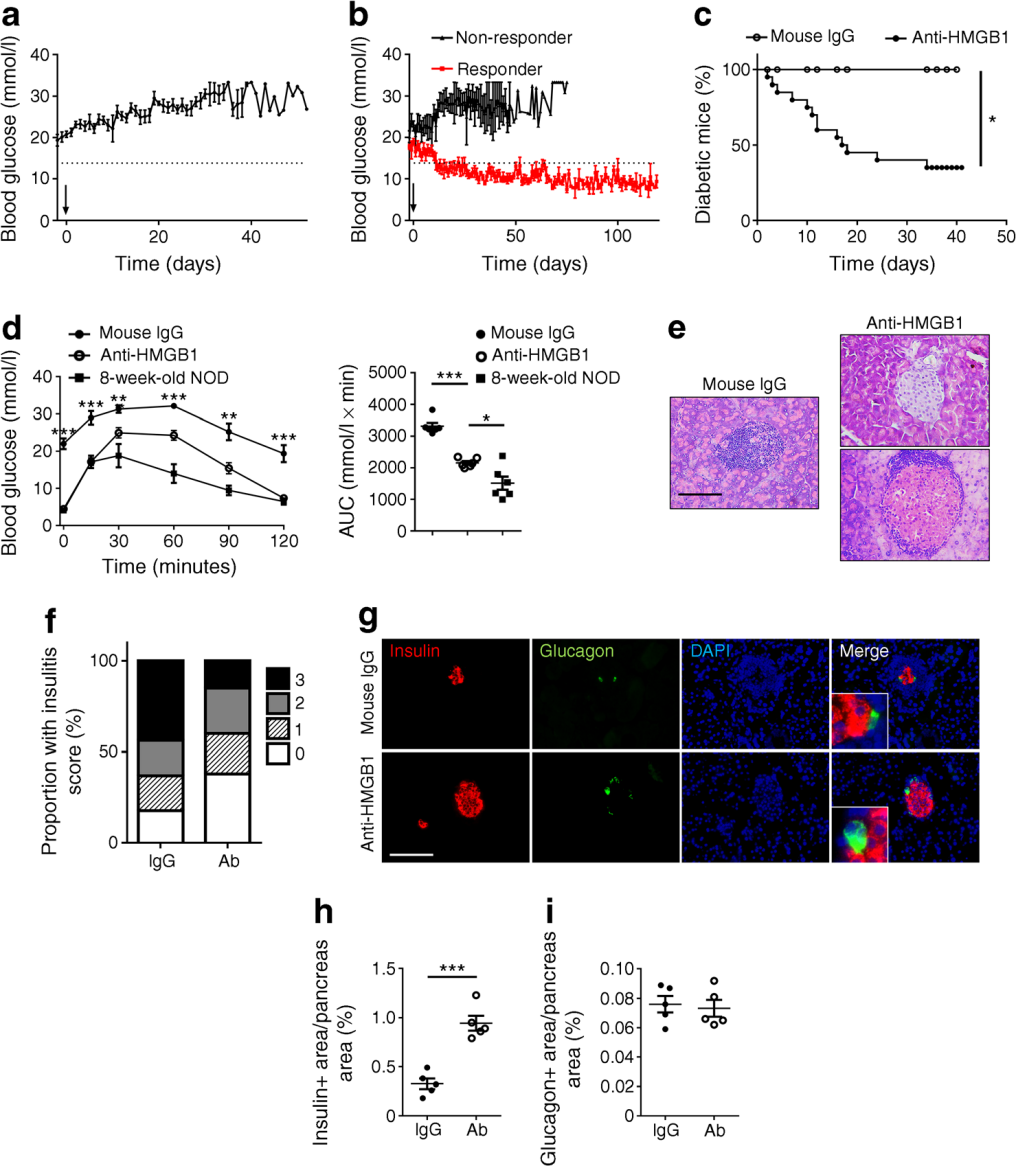
Anti-HMGB1 therapy results in the reversal of new-onset diabetes. (**a**) Blood glucose levels of mice in the control group (*n* = 10, 12–20 weeks of age). The arrow indicates the start time of mouse IgG treatment. (**b**) Blood glucose levels in anti-HMGB1-treated mice (*n* = 20, 12–20 weeks of age; *n* = 7 for non-responders and *n* = 13 for responders). The arrow indicates the start time of neutralising antibody administration. (**c**) The proportion of mice that remained diabetic after treatment with mouse IgG (*n* = 10) or anti-HMGB1 (*n* = 20). (**d**) Results of intraperitoneal glucose tolerance tests and the AUC for blood glucose levels (*n* = 6 for each group). The 8-week-old mice were untreated prediabetic controls. (**e**) Representative H&E staining of pancreatic sections obtained from diabetic mice treated with mouse IgG or anti-HMGB1. (**f**) Insulitis score of the indicated groups. (**g**–**i**) Representative staining patterns of insulin and glucagon (**g**) and quantification of insulin- (**h**) and glucagon- (**i**) positive areas in diabetic mice after treatment with control IgG or anti-HMGB1 antibody. *n* = 5 per group (**e**–**i**). Scale bars, 100 μm (**e**, **g**). Boxed areas show magnified fields (**g**), and images were taken under ×400 magnification (**e**, **g**). Values are expressed as mean ± SEM. Statistical difference in (**c**) was analysed by a logrank test; in (**f**) was determined by the χ^2^ test (*p* < 0.001); in (**d**) the comparison for IgG vs anti-HMGB1 at each time point was by unpaired Student’s *t* test; and in other figure parts was analysed by unpaired Student’s *t* test; *p < 0.05, **p < 0.01, ***p < 0.001

Next, we assessed whether blockade of HMGB1 could reverse new-onset autoimmune diabetes. Once diagnosed with diabetes, the mice were treated with either the anti-HMGB1 (500 μg/mouse) or mouse IgG every other day for two weeks. None of the control IgG-treated mice exhibited a sustained decline in blood glucose levels (Fig. 2a). In striking contrast, anti-HMGB1 reversed new-onset autoimmune diabetes for at least 120 days in a majority of the mice (13 out of 20) (Fig. 2b,c). Although some of the 13 responders did not achieve normoglycaemia during the early stage of treatment, their blood glucose levels showed a decreasing but fluctuating trend and eventually fell below 13.8 mmol/l. Particularly, the responders were characterised by the significantly improved glucose tolerance (1 week after anti-HMGB1 therapy), although there was a slight decrease compared with the prediabetic 8-week-old NOD mice (Fig. 2d). Indeed, histological analysis of pancreatic sections derived from the responders (1 week after anti-HMGB1 therapy) revealed that there were more islets free of insulitis or with minimal infiltration in the form of peri-insulitis (Fig. 2e,f). Again, the control IgG-treated mice manifested higher severity of immune infiltration in the islets (Fig. 2e,f). Moreover, the anti-HMGB1-treated mice had structured islets with abundant insulin-positive cells, while more shrunk islets and fewer beta cells were noted in control IgG-treated mice (Fig. 2g). Similarly, a significantly increased insulin-stained area (Fig. 2h) along with an unaltered glucagon-stained area (Fig. 2i) were detected in the antibody-treated mice. Of note, no insulin and glucagon double-positive cells were found in either group, which excluded the possibility of transdifferentiation from alpha cells to beta cells after treatment. Together, our results support that neutralising HMGB1 reversed new-onset diabetes, decreased insulitis and preserved insulin-staining islets in diabetic NOD mice.

### IAA positivity and the starting blood glucose levels at the study entry is prerequisite to the success of anti-HMGB1 therapy

**Fig. 3:**
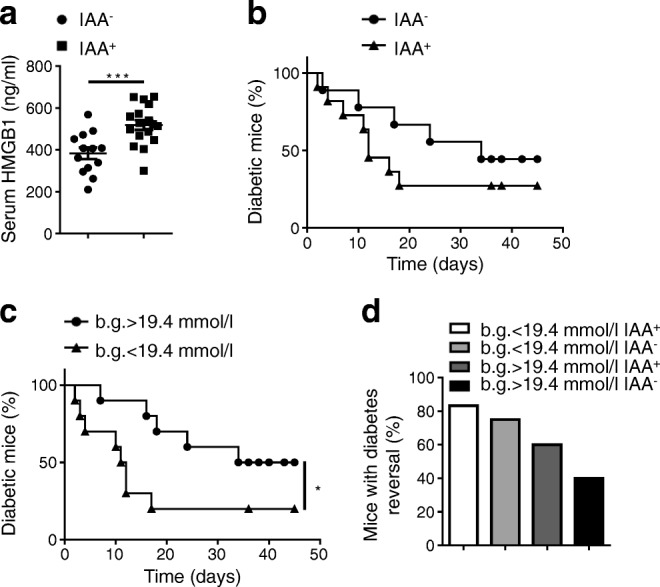
Blood glucose and IAA positivity at study entry predict the therapeutic success of anti-HMGB1. (**a**) Analysis of serum HMGB1 levels between IAA^−^ (*n* = 13) and IAA^+^ (*n* = 17) mice. (**b**) NOD mice with new-onset diabetes were stratified based on the presence of IAAs at the study entry. The incidence of diabetes is shown as the percentage of mice that remained diabetic after anti-HMGB1 therapy; *n* = 9 for IAA^−^ and *n* = 11 for IAA^+^. (**c**) NOD mice with new-onset diabetes were stratified based on starting blood glucose concentrations (b.g.) less than or more than 19.4 mmol/l at the study entry. Incidence of diabetes is shown as in (**b**); *n* = 10 per group. (**d**) New-onset diabetic NOD mice were stratified based on the starting b.g. and IAA status at study entry and the percentage of mice with disease reversal after therapy is shown; *n* = 6 for b.g. <19.4 mmol/l IAA^+^, *n* = 4 for b.g. <19.4 mmol/l IAA^−^, *n* = 5 for b.g. > 19.4 mmol/l IAA^+^ and *n* = 5 for b.g. > 19.4 mmol/l IAA^−^. Values are expressed as mean ± SEM. Statistical difference in (**a**) was analysed by unpaired Student’s *t* test; in (**b**, **c**) was compared by a logrank test; in (**d**) was determined by the χ^2^ test; *p < 0.05, ***p < 0.001

To address why diabetes reversal did not occur in some mice treated with anti-HMGB1 therapy, we first examined serum IAA and HMGB1 levels in the above ten control mice and 20 mice treated with anti-HMGB1. Of note, compared with IAA^−^ mice, IAA^+^ mice displayed significantly higher levels of serum HMGB1 (Fig. 3a). We then compared the success of anti-HMGB1 therapy between IAA^+^ and IAA^−^ mice, and interestingly found that IAA positivity at the study entry seemed to be positively correlated with therapeutic success (Fig. 3b). Next, we assessed the difference in anti-HMGB1 therapy between mice with blood glucose levels >19.4 mmol/l and <19.4 mmol/l at study entry. Remarkably, mice with blood glucose <19.4 mmol/l displayed significantly higher diabetes reversal rate (Fig. 3c). Finally, we assessed the therapeutic effect of HMGB1-neutralising antibody by combining IAA positivity and blood glucose levels at study entry. Mice with blood glucose <19.4 mmol/l and IAA positivity at the start of neutralising antibody administration had an apparently superior diabetes remission rate (83%; *n* = 6) than that of mice with blood glucose >19.4 mmol/l but absence of IAA (40%; *n* = 5), although this difference was not statistically significant (Fig. 3d). Collectively, our data support the idea that initial blood glucose levels and IAA positivity could be markers to predict the therapeutic outcome for reversal of autoimmune diabetes following anti-HMGB1 therapy.

### Anti-HMGB1 therapy prevents the recurrence of autoimmunity following syngeneic islet transplantation

There is compelling evidence that a large amount of HMGB1 is released from islets soon after their transplantation, which then initiates the eventual early loss of transplanted islets [25–27], and a similar effect was also observed in clinical settings [28]. Therefore, we next examined whether neutralising HMGB1 would prevent the recurrence of autoimmunity and prolong islet isograft survival in NOD mice.

**Fig. 4:**
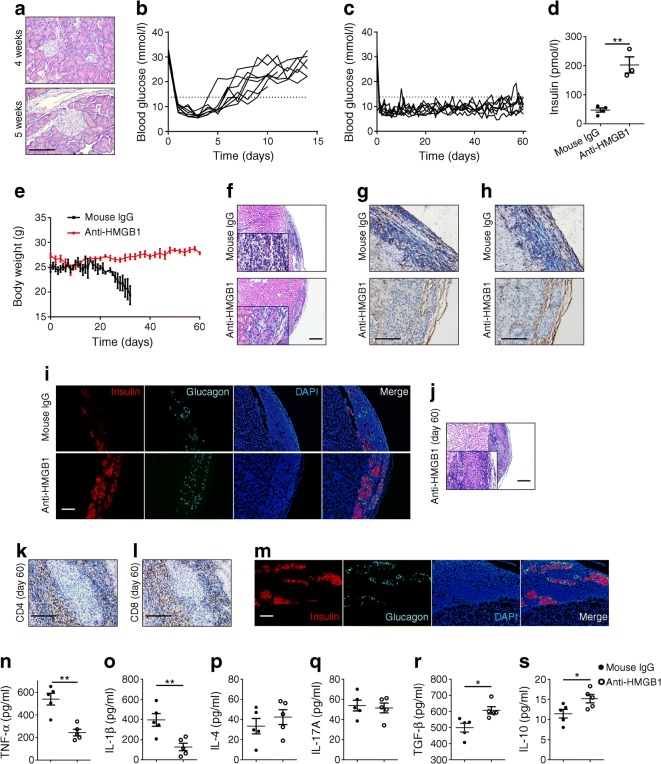
Islet isograft survival is prolonged in diabetic NOD mice by targeting HMGB1. (**a**) H&E staining of pancreatic sections obtained from 4- or 5-week-old NOD mice (representative of *n* = 6 per group). (**b**) Blood glucose levels in islet graft-receiving mice treated with control IgG (*n* = 8). (**c**) Blood glucose levels in islet graft-receiving mice treated with anti-HMGB1 (*n* = 8). (**d**) Serum insulin levels determined 9 days after islet transplantation; *n* = 4 for IgG-treated mice and *n* = 3 for anti-HMGB1-treated mice. (**e**) Comparison of body weight changes between control IgG- and anti-HMGB1-treated mice after islet transplantation; *n* = 8 per group. (**f**) Representative results showing H&E staining of islet grafts in IgG-treated NOD mice or anti-HMGB1-treated NOD mice 9 days after transplantation. (**g**, **h**) Representative immunohistochemical staining for CD4 (**g**) and CD8 (**h**) (brown staining) in graft sections at day 9 after islet transplantation. (**i**) Representative double staining for insulin and glucagon in islet grafts 9 days after transplantation. (**j**) Representative H&E staining of islet grafts obtained from anti-HMGB1-treated NOD mice 60 days after transplantation. (**k**, **l**) Representative immunohistochemical staining for CD4 (**k**) and CD8 (**l**) (brown staining) in graft sections obtained from anti-HMGB1-treated mice 60 days after transplantation. (**m**) Representative double staining for insulin and glucagon in islet grafts obtained from the anti-HMGB1-treated group 60 days after transplantation. (**n**–**s**) Analysis of serum cytokine levels 9 days after islet transplantation. *n* = 4 per group (**f**–**m**); *n* = 5 per group (**n**–**s**). Scale bars, 200 μm (**f**, **j**); 100 μm (**a**, **g**–**i**, **k**–**m**); original magnification ×100 (**f**, **j**); ×200 (**i**, **m**); ×400 (**a**, **g**, **h**, **k**, **l**). Values are expressed as mean ± SEM. Statistical difference was analysed by unpaired Student’s *t* test; *p < 0.05, **p < 0.01

Islets were isolated from 4- to 5-week-old NOD mice as there was no obvious lymphocyte infiltration at this stage (Fig. 4a). Given that anti-HMGB1 treatment reversed newly diagnosed diabetes, spontaneous diabetic NOD females were treated with sub-therapeutic doses of insulin for at least 4 weeks prior to transplantation, and by then almost no functional beta cells could be detected (ESM Fig. 2). Five hundred islets were next implanted into the left kidney capsule. The recipient mice were randomly grouped to receive anti-HMGB1 therapy or control IgG every other day starting at one day before transplantation for 2 weeks. As expected, the control IgG-treated mice rapidly lost their islet grafts and returned to a hyperglycaemic state 9 days (the median survival period) after transplantation (Fig. 4b). Notably, anti-HMGB1 therapy significantly prolonged graft survival as manifested by the maintenance of normal blood glucose levels (Fig. 4c). In line with the observed restoration of normoglycaemia, insulin deficiency (Fig. 4d) and diabetes-induced body weight loss (Fig. 4e) were abolished in anti-HMGB1-treated mice. Together, these data suggest that neutralising HMGB1 protected NOD mice against recurrent autoimmune attack after islet transplantation.

Islet grafts on day 9 following transplantation (the median time point associated with diabetes recurrence) were subjected to H&E staining and immunohistochemical analysis. Loss of islet architecture (Fig. 4f) and severe inflammatory infiltration throughout the grafts (Fig. 4g,h) along with loss of most insulin-positive cells (Fig. 4i) were observed in control IgG-treated mice. In contrast, anti-HMGB1-treated recipients showed intact islet mass (Fig. 4f) and almost complete absence of inflammatory infiltration (Fig. 4g,h) along with functional beta cells (Fig. 4i). Importantly, even after day 60 of transplantation, the majority of grafts still manifested intact islet mass (Fig. 4j), and immune infiltration was limited to the periphery of grafts (Fig. 4k,l) with well-preserved functional beta cells (Fig. 4m). Consistent with these observations, anti-HMGB1 therapy substantially decreased serum levels for IL-1β and TNF-α, and increased TGF-β and IL-10 on day 9 of transplantation, but without perceptible impact on IL-4 and IL-17A levels (Fig. 4n–s). Together, anti-HMGB1 therapy preserved islet graft mass and attenuated autoimmune infiltration.

### Blockade of HMGB1 expands the CD4^+^FOXP3^+^ Treg compartment and affects T cell subpopulations

**Fig. 5:**
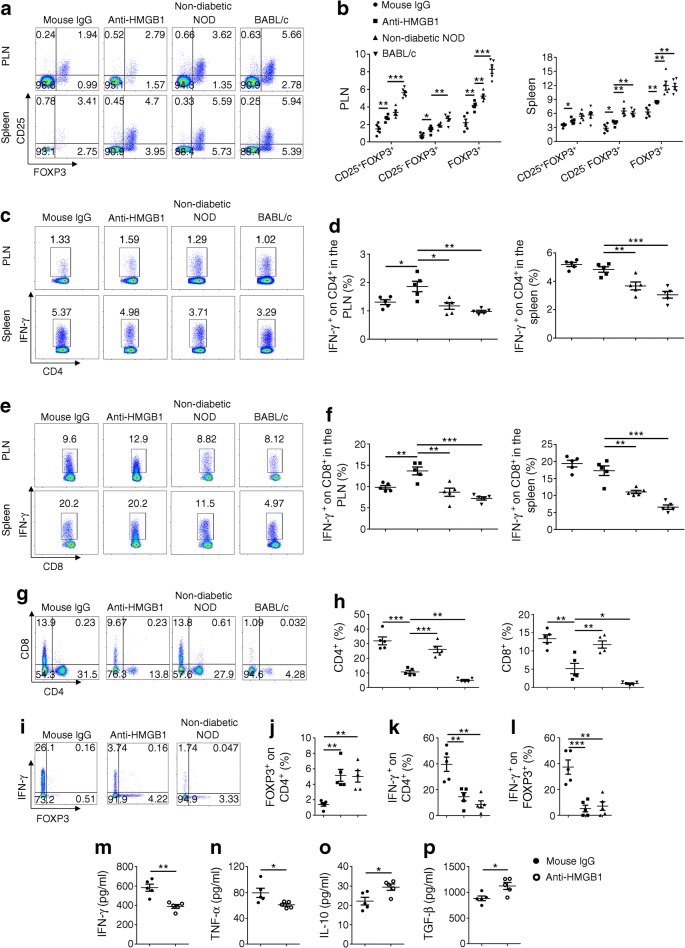
Blockade of extracellular HMGB1 regulates T cell subsets. (**a**, **b**) Representative FACS plots (**a**) and frequencies (**b**) of CD25^+^FOXP3^+^ cells, CD25^−^FOXP3^+^ cells and total FOXP3^+^ cells gated on CD4^+^ T cells in diabetic mice that received mouse IgG or anti-HMGB1, non-diabetic NOD mice and BALB/c mice. Numbers in quadrants indicate percentages of cells. (**c**, **d**) Representative FACS plots (**c**) and frequencies (**d**) of IFN-γ expression by CD4^+^ T cells in diabetic mice given mouse IgG or anti-HMGB1, non-diabetic NOD mice and BALB/c mice. (**e**, **f**) Representative FACS plots (**e**) and frequencies (**f**) of IFN-γ expression by CD8^+^ T cells in diabetic mice treated with mouse IgG or anti-HMGB1, non-diabetic NOD mice and BALB/c mice. (**g**, **h**) Representative FACS plots (**g**) and frequencies (**h**) of CD4^+^ and CD8^+^ T cells in pancreas. (**i**–**l**) Representative FACS plots (**i**) and the percentages of FOXP3^+^ cells (**j**) and IFN-γ^+^ cells (**k**) within the CD4^+^ T cell population and IFN-γ^+^ on FOXP3^+^ cells (**l**) in the pancreas. (**m**–**p**) Cytokine profile of culture supernatants of PLN mononuclear cells obtained from anti-HMGB1- or mouse IgG-treated diabetic mice. *n* = 5 per group. The key above (**b**) applies to (**d**, **f**, **h**, **j**–**l**; note that BALB/c was not used in **i**–**l**). Values are expressed as mean ± SEM. Statistical difference was analysed by unpaired Student’s *t* test; *p < 0.05, ** *p* < 0.01, ***p < 0.001

To dissect the mechanisms underlying the protective effects of anti-HMGB1 therapy described above, we first examined the impact of anti-HMGB1 treatment on Treg production in newly diabetic mice. The proportion of CD4^+^ forkhead box P3 (FOXP3)^+^ (both CD25^+^ and CD25^−^) T cells was significantly higher in the spleen and PLNs of anti-HMGB1-treated mice than those of IgG-treated mice (Fig. 5a,b). Next, we examined the proportions of CD4^+^IFN-γ^+^ (Th1) and CD8^+^IFN-γ^+^ (Tc1) cells. Interestingly, the proportions of PLN Th1 (Fig. 5c,d) and Tc1 (Fig. 5e,f) cells were significantly higher in anti-HMGB1-treated animals than IgG-treated mice. In contrast, there were no significant differences in the proportions of splenic Th1 and Tc1 cells between the two groups of mice. We then checked CD4^+^ and CD8^+^ T cell subpopulations in the pancreas, and found that CD4^+^ and CD8^+^ T cells were lower in anti-HMGB1-treated mice compared with both IgG-treated mice and non-diabetic NOD mice (Fig. 5g,h). Importantly, the proportion of Tregs was significantly higher but remarkably lower for Th1 cells in anti-HMGB1-treated mice (Fig. 5i–k), and anti-HMGB1 therapy substantially reduced the proportion of IFN-γ^+^FOXP3^+^ cells within the Treg population in the pancreas, which was similar to the percentage observed in non-diabetic mice (Fig. 5i,l).

Next, we examined the cytokine profiles of culture supernatants from PLN mononuclear cells. Anti-HMGB1 treatment suppressed IFN-γ and TNF-α secretion but enhanced IL-10 and TGF-β secretion (Fig. 5m–p) without discernible impact on IL-4 and IL-17A secretion (ESM Fig. 3). Altogether, our data support the suggestion that blockade of HMGB1 attenuated the migration of effector T cells from PLNs to the pancreatic islets and shifted the cytokine balance from a Th1 pattern towards an anti-inflammatory profile.

### HMGB1 impairs Treg stability and functionality

**Fig. 6:**
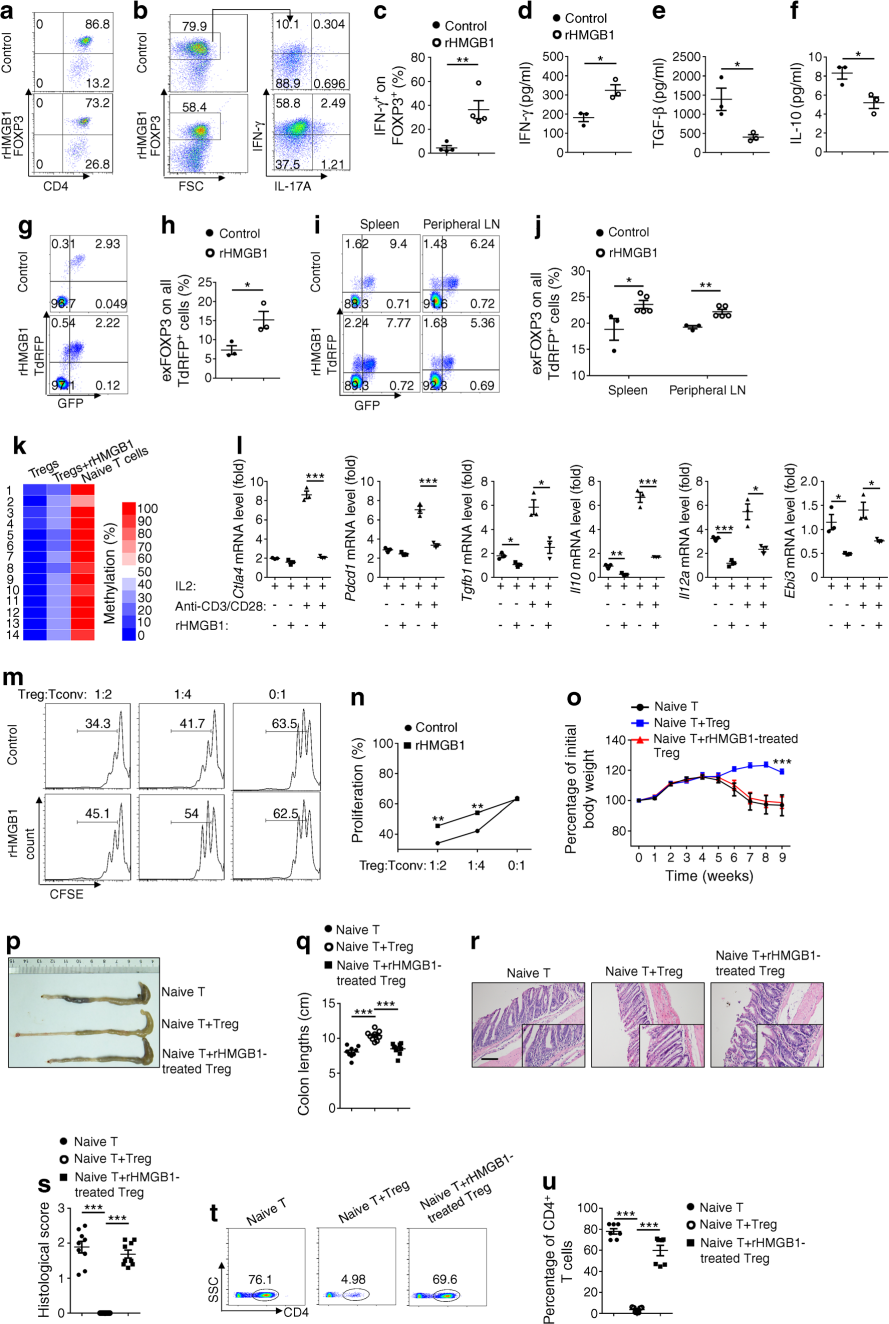
rHMGB1 exerts negative effect on Treg characteristics. (**a**) Frequency of FOXP3^+^CD4^+^ T cells after exposure of naive CD4^+^ T cells to Treg-inducing conditions with or without rHMGB1. (**b**, **c**) Isolated Tregs were cultured with or without rHMGB1 for 3 days. Representative FACS plots (**b**) and frequencies (**c**) of IFN-γ or IL-17A expression by CD4^+^FOXP3^+^ T cells. (**d**–**f**) Cytokine profile of culture supernatants of isolated Tregs. (**g**, **h**) Splenocytes were isolated from transgenic reporter mice and cultured with or without rHMGB1 for 3 days. Representative FACS plots (**g**) and frequencies (**h**) of exFOXP3 cells. (**i**, **j**) Representative FACS plots (**i**) and frequencies (**j**) of exFOXP3 cells (among CD4^+^ cells) from the spleen and peripheral lymph nodes of transgenic reporter mice 1 week after rHMGB1 intraperitoneal administration (120 μg/kg; *n* = 3 for control and *n* = 5 for rHMGB1 treatment). (**k**) Methylation status of CpG motifs of the TSDR at the *Foxp3* locus, assessed by bisulphite sequencing of isolated Tregs activated with anti-CD3/CD28 and IL-2 for 72 h in the presence of rHMGB1. Numbers on the left (1–14) indicate the 14 CpG islands from 5′ to 3′ in the intron 1 of the *Foxp3* locus. (**l**) Relative gene expression levels in isolated Tregs stimulated with or without rHMGB1 and anti-CD3/CD28 for 12 h in the presence of IL-2. (**m**) CD4^+^ Tconv were labelled with carboxyfluorescein diacetate succinimidyl ester (CFSE), stimulated with plate-coated anti-CD3/CD28 and cultured alone or with rHMGB1-pretreated Tregs at the indicated ratios. CFSE dilution was measured by flow cytometry after 3 days. Histograms depict cellular proliferation and were gated on viable cells. (**n**) Summary of the experiments involving Treg:Tconv ratios, as indicated. (**o**) Body weight changes after naive T cell adoptive transfer into NOD-*scid* host mice alone or in combination with Tregs. (**p**) Representative images of the colons of host mice 9 weeks after transfer and (**q**) graph showing colon lengths in the different groups. (**r**) Representative histological images of colon 9 weeks after transfer. (**s**) Graph summarising histological severity score. (**t**) FACS analysis of transferred T cells in the colon lamina propria. (**u**) Proportion of congenic CD4^+^ cells from the lamina propria of mice as in (**t**). *n* = 9 per group (**o**–**s**); *n* = 7 per group (**t, u**). Scale bar, 100 μm. Original magnification ×200. All in vitro studies were conducted 3 times. Values are presented as mean ± SEM. Statistical difference in (**n**) was determined by unpaired Student’s *t* test at each ratio; in (**o**) was for naive T + Treg vs naive T + rHMGB1-treated Treg in week 9 by unpaired Student’s *t* test; and in other figure parts was by unpaired Student’s *t* test; *p < 0.05, **p < 0.01, ***p < 0.001. LN, lymph node; TdRFP, tandem-dimer red fluorescent protein

The above results prompted us to assess the impact of HMGB1 on Treg characteristics. Under Treg-polarising conditions, addition of recombinant HMGB1 (rHMGB1) only slightly attenuated the differentiation of naive CD4^+^ T cells towards a Treg fate (Fig. 6a and ESM Fig. 4a). Then we enriched Tregs with a CD4^+^CD25^+^ Regulatory T Cell Isolation Kit and checked their purity (ESM Fig. 4b). Anti-CD3/28 combined with rHMGB1 stimulation led to the loss of FOXP3 expression in Tregs, paralleled by the acquisition of IFN-γ and, to a lesser extent, IL-17A expression (Fig. 6b,c). Moreover, IFN-γ levels were significantly higher, whereas IL-10 and TGF-β levels were lower in the culture supernatants (Fig. 6d–f). To further confirm the impact of HMGB1 on Treg stability, we employed transgenic reporter mice that carried a *Foxp3* lineage reporter (ESM Fig. 5) [29, 30]. In these mice, Tregs that express or have ever expressed FOXP3 are tomato red^+^, while Tregs that currently express FOXP3 are GFP^+^, and cells that have lost FOXP3 expression are GFP^−^. Thus, these ‘exFOXP3’ cells can be easily distinguished from functional Tregs. Remarkably, rHMGB1 stimulation significantly increased the frequency of exFOXP3 cells in vitro (Fig. 6g,h) and in vivo (Fig. 6i,j). In addition, methylation levels for TSDR, which is associated with the maintenance of FOXP3 expression and resultant Treg stability, was higher in rHMGB1-treated Tregs in vitro (Fig. 6k). Furthermore, rHMGB1 stimulation downregulated Treg function-related genes, especially in the presence of anti-CD3/28 (Fig. 6l).

A Treg suppression assay was next conducted to assess the impact of HMGB1 on Treg suppressive function. In comparison with the control Tregs, CD4^+^CD25^−^ T cells cultured with rHMGB1-stimulated Tregs showed markedly higher proliferative activities (Fig. 6m,n). To further confirm the impact of HMGB1 on Treg function in vivo, we used the well-established T cell-transfer model of colitis in NOD-*scid* mice. As expected, transfer of NOD naive CD4^+^ T cells alone led to a severe inflammation associated with progressive weight loss (Fig. 6o), along with colon shortening (Fig. 6p,q) and disrupted colon structure (Fig. 6r,s). In contrast, co-transfer of NOD Tregs, but not rHMGB1 pretreated NOD Tregs, prevented disease (Fig. 6o–s). Remarkably, FACS analysis further revealed that the percentage of congenic CD4^+^ T cells in the lamina propria was much higher in mice with co-transfer of rHMGB1 pretreated Tregs (Fig. 6t,u and ESM Fig. 6). Collectively, our data indicate that HMGB1 impairs Treg stability and functionality.

### HMGB1 impairs Treg stability by regulating RAGE/TLR4-dependent PI3K–Akt–mTOR signalling

**Fig. 7:**
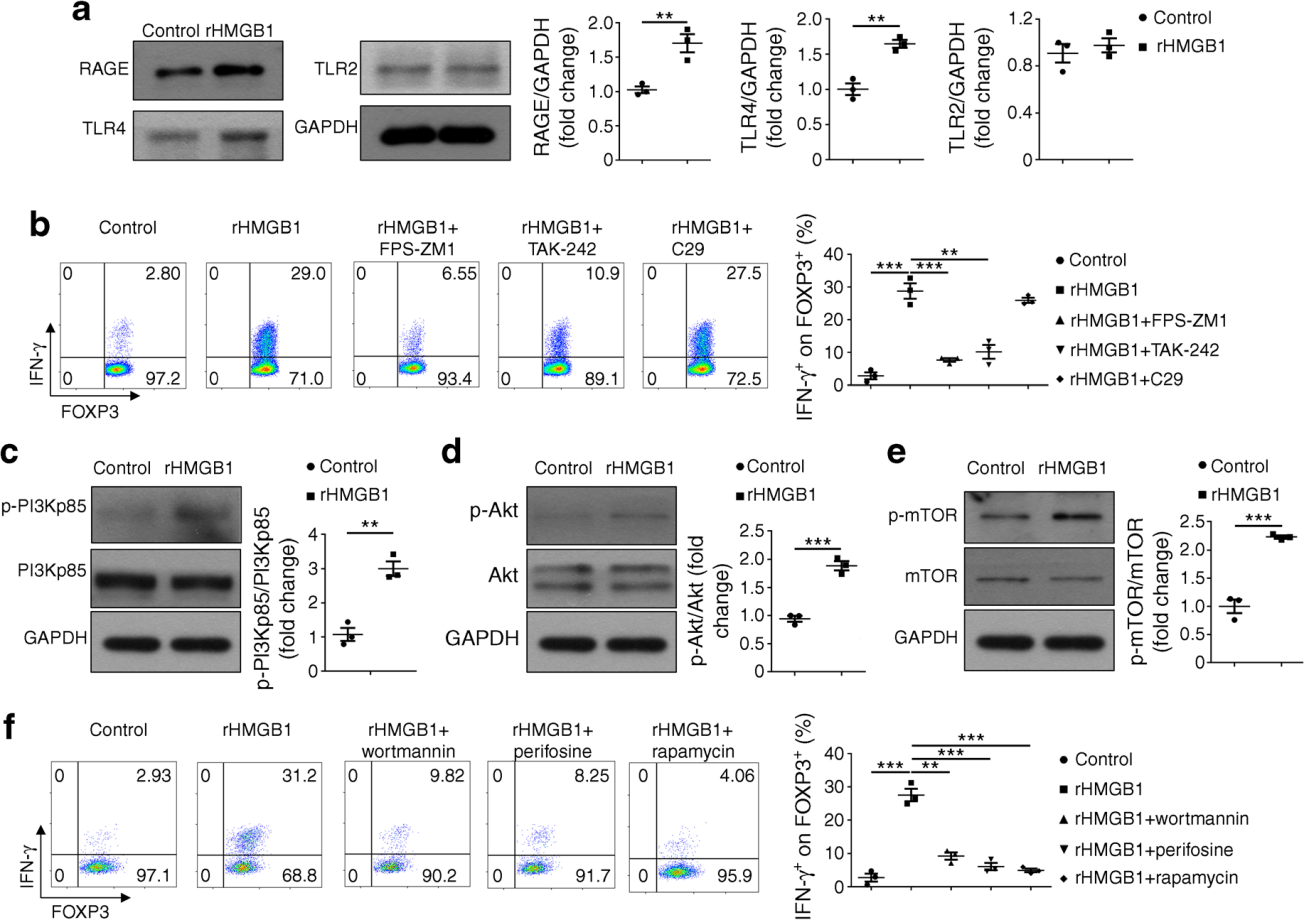
Upregulated PI3K–Akt–mTOR signalling contributes to the instability of rHMGB1-treated Tregs. (**a**) Representative western blot analysis and quantification of RAGE, TLR4 and TLR2 levels in Tregs after 3 days of rHMGB1 stimulation. (**b**) Representative FACS plots and frequency graph of IFN-γ expression by CD4^+^FOXP3^+^ T cells with the indicated inhibitors. (**c**–**e**) Representative western blot analysis and quantification of the following proteins in the PI3K–Akt–mTOR signalling pathway in Tregs treated with rHMGB1 for 24 h: p-PI3Kp85, PI3Kp85, p-Akt, Akt, p-mTOR and mTOR. (**f**) Representative FACS plots and frequency graph of IFN-γ expression by CD4^+^FOXP3^+^ T cells with the indicated inhibitors. All in vitro studies were conducted 3 times. Values are expressed as mean ± SEM. Statistical difference was analysed by unpaired Student’s *t* test; **p < 0.01, ***p < 0.001. C29, TLR2 inhibitor; FPS-ZM1, RAGE inhibitor; perifosine, Akt inhibitor; rapamycin, mTOR inhibitor; TAK-242, TLR4 inhibitor; wortmannin, PI3K inhibitor. GAPDH, glyceraldehyde-3-phosphate dehydrogenase

To address mechanisms by which HMGB1 impairs Treg stability, we first examined the expression of HMGB1 receptors in Tregs. It was noted that rHMGB1 induced a significant increase in the expression of receptor for AGE (RAGE) and toll-like receptor (TLR)4 but no perceptible change for TLR2 expression (Fig. 7a). More importantly, blockade of RAGE (FPS-ZM1) and TLR4 (TAK-242) signalling rescued Treg stability following rHMGB1 stimulation but not blockade of TLR2 (C29) signalling (Fig. 7b), suggesting that HMGB1 acts predominantly through the RAGE and TLR4 receptors. Next, we analysed RAGE/TLR4 downstream signalling. rHMGB1 induced a significant increase in phosphorylated phosphatidylinositol 3-kinase (PI3K) (Fig. 7c), Akt (Fig. 7d) and mechanistic target of rapamycin (mTOR) (Fig. 7e). To demonstrate that HMGB1 impairs Treg stability via RAGE/TLR4-dependent PI3K–Akt–mTOR signalling, wortmannin (a PI3K inhibitor), perifosine (an Akt inhibitor) and rapamycin (an mTOR inhibitor) were added together with rHMGB1 into the Treg cultures. Indeed, inhibition of PI3K–Akt–mTOR signalling attenuated rHMGB1-induced Treg instability (Fig. 7f). Collectively, our results demonstrate that rHMGB1 activates RAGE/TLR4 to enhance PI3K–Akt–mTOR signalling thereby impairing Treg stability.

### Circulating HMGB1 is associated with a higher IFN-γ^+^ Treg fraction in individuals with type 1 diabetes

**Fig. 8:**
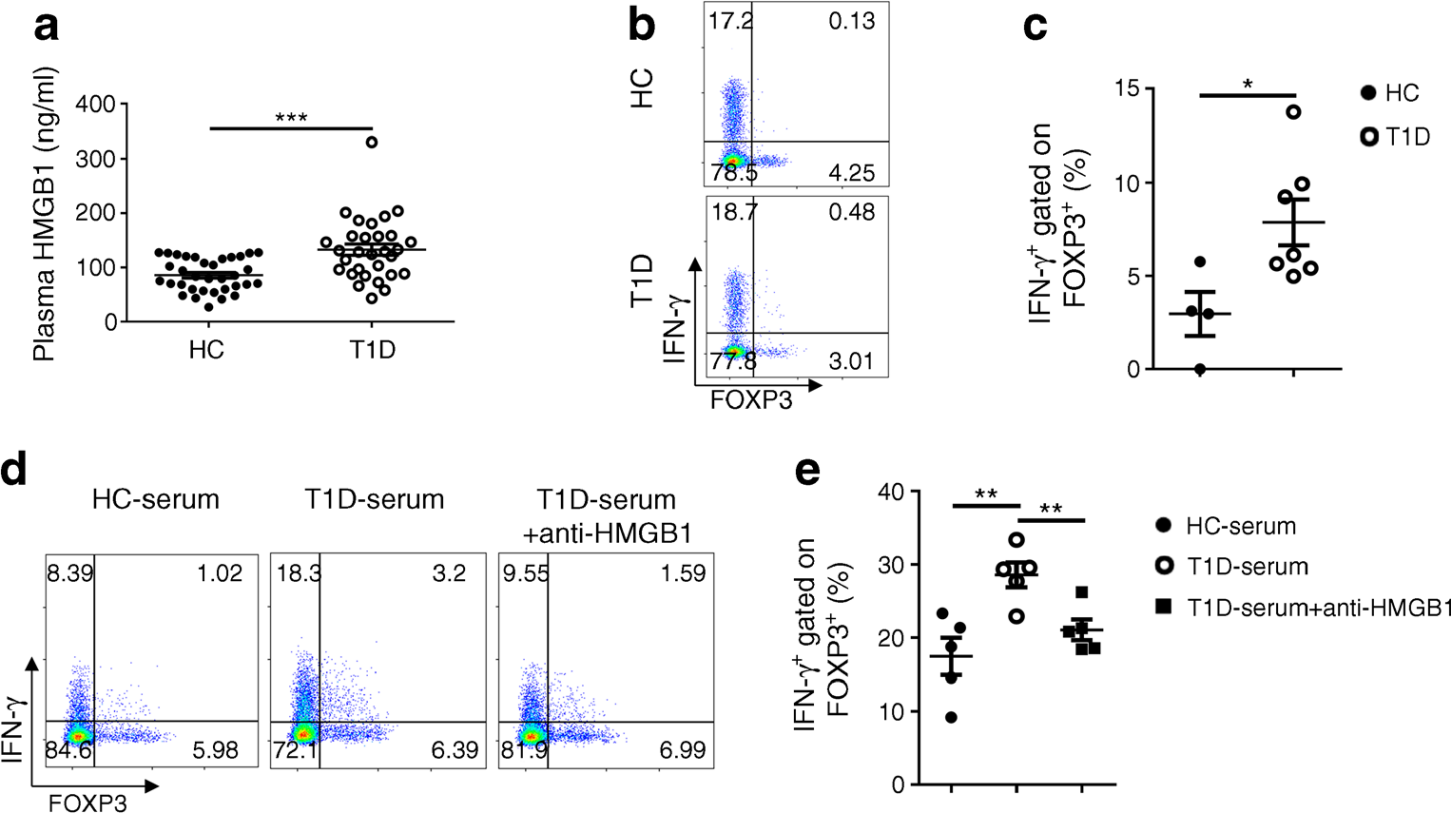
HMGB1 regulates peripheral IFN-γ^+^ Treg fraction in type 1 diabetes patients. (**a**) An elevated circulating HMGB1 level in participants with type 1 diabetes (*n* = 30) in comparison with healthy controls (*n* = 34). (**b**, **c**) CD4^+^ PBMCs were enriched and stimulated for 4 h with phorbol12-myristate 13-acetate (PMA)/ionomycin/GolgiStop. Representative flow cytometric analysis (**b**) and percentage (**c**) of IFN-γ and FOXP3 expression in the CD4^+^ gated population; *n* = 4 for control participants and *n* = 7 for diabetic participants. (**d**, **e**) PBMC and serum samples were freshly prepared from diabetic and control participants; *n* = 5 per group. HC-PBMCs were incubated with HC-serum or T1D-serum (final concentration: 20%) for 72 h in duplicate. HMGB1 was blocked or not using ant-HMGB1 during incubation of HC-PBMC with T1D-serum. Representative FACS plots (**d**) and frequency (**e**) of IFN-γ and FOXP3 expression by CD4^+^ T cells. Values are expressed as mean ± SEM. Statistical difference was analysed by unpaired Student’s *t* test; *p < 0.05, **p < 0.01, ***p < 0.001. HC, healthy control; T1D, type 1 diabetes

To explore the relevance of our data to human type 1 diabetes, we used ELISA to determine the concentration of plasma HMGB1 in 34 healthy control participants and 30 participants diagnosed with type 1 diabetes within a year (ESM Table 2). In line with our mouse data, individuals with diabetes manifested significantly higher levels of circulating HMGB1 than control participants (Fig. 8a). Interestingly, the frequency of Tregs in the peripheral blood was comparable (ESM Fig. 7a) but the proportion of IFN-γ^+^FOXP3^+^ Tregs was significantly higher in diabetic participants compared with control participants (Fig. 8b,c), which was consistent with previously reported data [31]. To further address the correlation between circulating HMGB1 levels and the proportion of IFN-γ^+^FOXP3^+^ Tregs in type 1 diabetic individuals, type 1 diabetic participants that had high levels of HMGB1 were selected. CD4^+^ peripheral blood mononuclear cells (PBMCs) isolated from heathy control participants were cultured with fresh serum originating from control and these type 1 diabetic participants, respectively. Although the proportion of Tregs was similar between two groups (ESM Fig. 7b), it was noted that serum derived from diabetic individuals induced significantly higher levels of IFN-γ^+^FOXP3^+^ Tregs, and blockade of HMGB1 by the neutralising antibody remarkably reduced the induction of IFN-γ^+^FOXP3^+^ Tregs (Fig. 8d,e), supporting the theory that circulating HMGB1 impairs Treg stability in individuals with type 1 diabetes.

## Discussion

In this report, we first provide experimental evidence indicating that HMGB1 is passively released from secondary necrotic beta cells during beta cell mass turnover in NOD mice. Previous studies [32, 33], including our own [15], have provided overall support for the notion that HMGB1 originating from secondary necrotic beta cells is a highly potent stimulator of DC activation. Given the critical role of DCs in type 1 diabetes-associated autoimmunity, we treated 2.5-week-old NOD mice with an HMGB1-blocking antibody. As expected, blockade of HMGB1 prevented insulitis progression and decreased diabetes incidence.

The initiation and effector phases of diabetes occur before the onset of hyperglycaemia and have the lowest threshold for disease prevention in NOD mice [34]. However, once autoimmune attack progresses along with the onset of hyperglycaemia, it becomes much more difficult to reverse diabetes and prevent recurrent autoimmunity following islet transplantation. As a result, almost no effective therapeutic strategies are available against the disease at these stages [34]. We thus sought to explore the therapeutic role of HMGB1 after overt diabetes occurs. For this purpose, two different models were employed: one aimed to reverse diabetes in new-onset diabetic NOD mice, while the other aimed to prevent recurrence of autoimmunity following syngeneic islet transplantation. Our initial results showed that blockade of HMGB1 was highly effective in reversing diabetes, as evidenced by inducing long-term normoglycaemia (>120 days). Similarly, neutralisation of HMGB1 remarkably prevented recurrent autoimmune attack of beta cells, as demonstrated by the preserved beta mass and attenuated severity of insulitis. Furthermore, it is likely that residual beta cell reserve (classed as initial blood glucose <19.4 mmol/l in our study) and IAA positivity could be predictive markers for the success of reversal diabetes. Generally, IAA^+^ diabetic mice are probably associated with higher severity of autoimmunity. Notably, IAA^+^ diabetic mice displayed higher circulating levels of HMGB1, and it is therefore logical that IAA^+^ diabetic mice could be in a more aggressive disease phase, which would render them more sensitive to anti-HMGB1 therapy. We also noted that HMGB1 neutralising antibody unexpectedly increased the percentage of Th1 and Tc1 cells in PLNs. Given the fact that anti-HMGB1 therapy significantly inhibited insulitis progression (characterised by the reduced islet immune infiltration), we believe that this phenomenon could be associated with repressed migration of activated autoreactive T cells from the PLN into the pancreatic islets. Indeed, blockade of HMGB1 resulted in a substantial immunosuppression evidenced by the increased CD4^+^FOXP3^+^ (both CD25^−^ and CD25^+^) Tregs in the PLN and spleen. In our second experimental model, we used an HMGB1 neutralising antibody to enhance long-term graft survival in a stringent model of isogeneic islet transplantation performed in hyperglycaemic NOD mice. Histological analysis of the grafts showed that islet morphology was preserved, and immunological analyses revealed that autoimmunity was suppressed in anti-HMGB1-treated mice.

To dissect the mechanisms by which anti-HMGB1 promotes Tregs in NOD mice, we first characterised the receptors of HMGB1 in Tregs, and then assessed the impact of HMGB1 on Treg characteristics. RAGE and TLR4 were found to be the predominant HMGB1 receptors in Tregs. The stability of Tregs was impaired after rHMGB1 stimulation, as displayed by the increased TSDR methylation levels in vitro along with the presence of IFN-γ^+^FOXP3^+^ Tregs, which manifest attenuated suppressive functions. Indeed, previous studies demonstrated the production of Th1-like Tregs in patients with autoimmune disease such as type 1 diabetes [31] and multiple sclerosis [35, 36], and in inflammatory environments with neurotropic hepatitis virus [37] and *Toxoplasma gondii* infection [38]. The negative regulatory effect of HMGB1 on Tregs has been demonstrated in other human diseases [39–42], but the molecular mechanisms underlying these effects have yet to be fully addressed. Remarkably, blockade of RAGE or TLR4 attenuated HMGB1-induced Treg instability, while rHMGB1 stimulation resulted in significantly higher levels of phosphorylated PI3K–Akt–mTOR in Tregs, indicating that RAGE and TLR4 are essential for the HMGB1 downstream pathways in Tregs. Finally, we found that high circulating levels of HMGB1 in human participants with type 1 diabetes contribute to Treg instability, which paves the way for its use in clinical settings. Of note, hyperglycaemia may also enhance HMGB1 secretion by other types of cells such as endothelial cells [43], which may in turn exacerbate autoimmune progression.

In summary, we have provided strong evidence indicating the involvement of HMGB1 in type 1 diabetes pathogenesis. Blockade of extracellular HMGB1 altered the course of the disease even after the autoimmune response had evolved into overt diabetes. The mechanisms underlying this protective effect are associated with the promotion of immunosuppression, especially via factors that maintain the stability and function of Tregs.

## Electronic supplementary material

ESM (PDF 752 kb)

## Data Availability

All data needed to evaluate the conclusion in the paper are present in the paper and/or the ESM. Additional data related to this paper may be requested from the authors.

## Abbreviations

DC: Dendritic cell
FOXP3: Forkhead box P3
HMGB1: High-mobility group box 1
IAA: Insulin autoantibody
mTOR: Mechanistic target of rapamycin
PBMC: Peripheral blood mononuclear cell
PI3K: Phosphatidylinositol 3-kinase
PLN: Pancreatic lymph node
RAGE: Receptor for AGE
rHMGB1: recombinant HMGB1
Tconv: conventional T cells
TLR: Toll-like receptor
Tregs: Regulatory T cells
TSDR: Treg cell-specific demethylated region
