# Supplementary material for: Extracellular HMGB1 exacerbates autoimmune progression and recurrence of type 1 diabetes by impairing regulatory T cell stability
Source: Diabetologia. 2020 Feb 19;63(5):987–1001. doi: 10.1007/s00125-020-05105-8 (PMC7145789; doi:10.1007/s00125-020-05105-8)
Supplement: Supplementary file 1 — (PDF 752 kb) [file 125_2020_5105_MOESM1_ESM.pdf]

Electronic supplementary materials (ESM)

Extracellular HMGB1 Exacerbates Autoimmune Progression and Recurrence of Type 1 Diabetes by impairing Regulatory T Cell Stability

Jing Zhang[1,2] (ORCID iD: 0000-0002-7346-555X), Longmin Chen[1,2] (ORCID iD: 0000-0002-9790-7949), Faxi Wang[1,2], Yuan Zou[1,2], Jingyi Li[1], Jiahui Luo[1,2], Faheem Khan[1], Fei Sun[1,2], Yang Li[1,2], Jing Liu[1,2], Zhishui Chen[1,3], Shu Zhang[1,2], Fei Xiong[1,2], Qilin Yu[1], Jinxiu Li[4], Kun Huang[5], Bao-Ling Adam[6], Zhiguang Zhou[7], Decio L. Eizirik[8], Ping Yang[1,2] and Cong-Yi Wang[1,2,3]

ESM Methods

Production and purification of an HMGB1 neutralising antibody

Hybridoma cells (2E11P) capable of secreting an HMGB1 monoclonal neutralising antibody were transferred from the Vanderbilt University (Nashville, Tennessee, USA). The cell line was routinely tested and authenticated negative for mycoplasma contamination. Briefly, hybridoma cells were injected intraperitoneally into BALB/c mice to induce the large-scale production of mAb. The antibody was purified by HiTrap Protein A HP (17-0402-01; GE, Piscataway, NJ, USA). To remove any contaminated endotoxin, mAbs were passed through Pierce High-Capacity Endotoxin Removal Spin Columns (88276; Pierce Biotechnology, Rockford, IL, USA) as instructed.

Measurement of insulin autoantibodies

Serum was collected from NOD mice with new-onset diabetes before treatment randomization. IAAs were measured using a Mouse IAA ELISA Kit (ml002189-J; mlbio, Shanghai, China), and analyzed as previously described [1].

ELISA

The protein levels of TNF-α, IFN-γ, IL-1β, IL-4, IL-17A, and IL-10 were determined using

the ELISA kits obtained from BD Biosciences (San Diego, CA, USA) with previously established techniques [2]. The level of TGF-β was determined using an ELISA kit obtained from eBioscience (San Diego, CA, USA). Serum insulin was measured using a Rat/Mouse Insulin ELISA kit (EZRMI-13K; Millipore, Billerica, Merck KGaA, USA). Circulating HMGB1 levels were detected in mice and humans using a Mouse/Rat HMGB1 ELISA Kit (ARG81310; arigo Biolaboratories, Hsinchu City, China) and a Human HMGB1/HMG-1 ELISA Kit (NBP2-62766; Novus Biologicals, CO, USA), respectively.

Histological and immunohistochemical analysis and immunofluorescence staining

Tissue sections were subjected to H&E staining [3], immunohistochemical staining (HMGB1, CD4 and CD8) [4], and immunofluorescence staining (insulin and glucagon) [5] as previously described. Insulitis scores were assessed in a blinded fashion by two examiners as follows: 0, normal islets; 1, peri-insulitis; 2, islets with lymphocyte infiltration in <50% of the area; and 3, islets with lymphocyte infiltration in >50% of the area or completely destroyed [6].

Flow cytometry

To evaluate surface markers, the cells were stained in PBS containing 2% BSA and FITC-conjugated anti-CD4 (557307), APC-Cy7-conjugated anti-CD8a (557654), PE-conjugated anti-CD25 (553075), PE-conjugated anti-CD44 (553134) and APC-conjugated anti-CD62L

(553152) antibodies. FOXP3 staining was conducted with APC-conjugated anti-FOXP3 (560401) antibodies according to the manufacturer's instructions for the Mouse FOXP3 Buffer Set obtained from BD Biosciences (560409; San Diego, CA, USA). To analyze intracellular cytokine expression, cells were stimulated with ionomycin (1 mM), phorbol12-myristate 13-acetate (PMA) (50 ng/ml), and GolgiStop for 4–6 h. At the end of stimulation, the cells were stained with PE-Cy7-conjugated IFN-γ (505826) and PE-conjugated IL-17A (559502) antibodies according to the manufacturer's instructions for Intracellular Staining Permeabilization Wash Buffer (421002; Biolegend, San Diego, CA, USA) [2]. The antibody against IFN-γ was purchased from eBioscience (San Diego, CA, USA), while all other antibodies were ordered from BD Biosciences (San Diego, CA, USA).

Cell purification and culture

To induce naïve $CD4^+$ T cell differentiating into $FOXP3^+$ cells, the isolated naïve cells (5×10^5 cells per ml) were seeded into plates coated with 10 ng/ml anti-CD3 and 10 ng/ml anti-CD28 antibodies, supplemented with 5 ng/ml TGF-β and 10 ng/ml IL-2 (R&D Systems, Minneapolis, MN, USA) for 3 days, and then the populations of differentiated cells were expanded for up to an additional 2 days in the presence of 5 ng/ml TGF-β and 10 ng/ml IL-2 [7]. The impact of the HMGB1–RAGE/TLRs–PI3K–AKT–mTOR axis on Treg stability was determined by applying inhibitors, including 1μM wortmannin (Santa Cruz Biotechnology, Santa Cruz, CA, USA), 4 μM perifosine (Selleck Chemicals, Houston, TX, USA), 100nM rapamycin (HY-10219; MedChemExpress, Shanghai, China),

500 nM FPS-ZM1 (HY-19370; MedChemExpress), 100 μM C29 (HY-100461; MedChemExpress), and 100nM TAK-242 (HY-11109; MedChemExpress), with the addition of human recombinant HMGB1 (rHMGB1) at 500ng/ml (1690-HMB-050; R&D Systems, Minneapolis, MN, USA).

Methylation analysis of TSDR

Briefly, enriched Tregs were cultured with anti-CD3/CD28 and IL-2 for 3 days in the presence of 500ng/ml rHMGB1 or not, and genomic DNA was prepared by a DNeasy Blood and Tissue Kit (69504; Qiagen, Redwood, CA, USA). Bisulfite conversion of DNA was conducted with a Methylamp™ DNA Modification Kit (P-1001-1; Epigentek, Farmingdale, NY, USA) as instructed. Intron 1 of *Foxp3* (corresponding to CNS2) was amplified with a primer set as described (forward, 5′-TAT TTT TTT GGG TTT TGG GAT ATT A-3′ and reverse, 5′-AAC CAA CCA ACT TCC TAC ACT ATC TAT-3′). PCR products were ligated into pGEM-T Easay vectors (A1380; Promega, Madison, WI, USA) and sequenced (10 sequences per sample).

Real-time PCR and Western blot analysis

Total RNAs were extracted from Tregs using the RNAiso plus reagents (9109; TaKaRa, Japan) as instructed. Real-time PCR was conducted to assess Treg function-related genes using an ABI prism 7500 Sequence Detection System (Applied Biosystems, San Francisco,

CA, USA). *Actb* was used for normalization, and the relative expression levels for each target gene were calculated using the $2^{-\Delta\Delta Ct}$ method.

For Western blot analysis, Treg lysates were separated on 10% SDS-PAGE gels and transferred onto PVDF membranes (Bio-Rad Laboratories, Hercules, CA, USA). The membranes were blocked with 5% nonfat milk for 1 h and then incubated with primary antibodies overnight at 4°C. Antibody against RAGE (1:1000 dilution, ab3611) were purchased from Abcam (Cambridge, MA, USA); antibodies against TLR2 (1:100 dilution, sc-10739), TLR4 (1:100 dilution, sc-16240), and GAPDH (1:100 dilution, sc-25778) were ordered from Santa Cruz Biotechnology (Santa Cruz, CA, USA); and antibodies against PI3K (1:1000 dilution, 4257s), phosphorylated PI3K (p-PI3K, Tyr458) (1:1000 dilution, 4228s), AKT (1:1000 dilution, 4685s), phosphorylated AKT (p-AKT, Ser473) (1:1000 dilution, 4060s), mTOR (1:1000 dilution, 2983s), and phosphorylated mTOR (1:1000 dilution, 2971s) were derived from Cell Signaling Technology (Danvers, MA, USA). After incubation with an HRP-conjugated secondary antibody, the reactive bands were visualized using an enhanced chemiluminescence system as reported [8].

In vitro suppression assays and T cell-transfer model of colitis

In vitro suppression assays:

$CD4^+CD25^-$ cells (conventional T cells) and $CD4^+CD25^+$ cells were purified to obtain

responder cells and Tregs, respectively. Tregs were preincubated with rHMGB1 (500 ng/ml) for 24 h. CFSE-labeled $CD4^+CD25^-$ cells were cultured in triplicates in round-bottom 96-well plates along with anti-CD3/CD28 stimulation. The inhibitory effect of Tregs on the proliferation of responder cells was tested by directly addition different ratios of Tregs into 2×10^5 responder cells. After 3 days of co-culture, cell proliferation was assessed by flow cytometry following CFSE dilution.

T cell-transfer model of colitis:

Colitis in NOD-scid mice was induced. Briefly, $CD4^+CD25^+$ Tregs and naïve $CD4^+CD44^{low/-}CD62L^+CD25^-$ T cells from NOD mice were prepared with isolation kits. Tregs were preincubated with or without rHMGB1 (500ng/ml) for 24 h and then washed in sterile PBS. Each NOD-scid recipient mice was given by intravenous injection of 2×10^5 naïve T cells alone or together with 5×10^4 Tregs. Body weight of mice was monitored weekly. The severity of colonic inflammation was graded by two pathologists in a blinded fashion using well-described four-point scales [9]: 0, normal tissue; 1, one or a few multifocal mononuclear cell infiltration in the lamina propria, along with minimal epithelial hyperplasia and slight to no mucus depletion from goblet cells; 2, more frequent lesions, and typical changes including several multifocal, mild inflammatory cell infiltration in the lamina propria. Mucin depletion and mild epithelial hyperplasia are also present. Inflammation rarely involves the submucosa and small epithelial erosions are occasionally observed; 3, lesions are more frequent or involve a large area of the mucosa. Inflammation is moderate and

usually involves the submucosa but is rarely transmural. Crypt abscesses are sometimes seen, and inflammatory cells are a mixture. Moderate epithelial hyperplasia and mucin depletion are present. Ulcers are accidently observed; 4, lesions often involve majority of the intestinal section. Inflammation is intense, and is sometimes transmural. Few mucin-containing cells and epithelial hyperplasia characterized by crowding of epithelial cells in elongated glands are present. Ulcers and crypt abscesses are seen and foci of fibrinoid necrosis are present in the submucosa contiguous to crypt abscesses and ulcerations.

ESM Results

Purification and confirmation of the HMGB1 neutralising antibody

We first purified the HMGB1-neutralising antibody from peritoneal dropsy obtained from BALB/c mice using protein A columns. The purified antibody was further passed over poly-lysine columns to remove any contaminating endotoxin. The purity of the antibody was high as determined by SDS-PAGE (ESM Fig. 1a). The purified monoclonal antibody showed high specificity and titers against HMGB1, as shown on the western blots (ESM Fig. 1b). To test its neutralising potency, rHMGB1 was added into the RAW264.7 cell cultures, which increased TNF-α secretion by 20-fold, while addition of above purified antibody 2 h before rHMGB1 stimulation, TNF-α secretion was reduced by 3-fold (ESM Fig. 1c), indicating that the neutralising antibody is highly potent to block the HMGB1 stimulatory effect.

References:

[1] Wiede F, Brodnicki TC, Goh PK, et al. (2019) T-Cell-Specific PTPN2 Deficiency in NOD Mice Accelerates the Development of Type 1 Diabetes and Autoimmune Comorbidities. Diabetes 68(6): 1251-1266. 10.2337/db18-1362

[2] Zhong J, Yu Q, Yang P, et al. (2014) MBD2 regulates TH17 differentiation and experimental autoimmune encephalomyelitis by controlling the homeostasis of T-bet/Hlx axis. Journal of autoimmunity 53: 95-104. 10.1016/j.jaut.2014.05.006

[3] Wei J, Long L, Yang K, et al. (2016) Autophagy enforces functional integrity of regulatory T cells by coupling environmental cues and metabolic homeostasis. Nat Immunol 17(3): 277-285. 10.1038/ni.3365

[4] Wang Y, Zhu J, Zhang L, et al. (2017) Role of C/EBP homologous protein and endoplasmic reticulum stress in asthma exacerbation by regulating the IL-4/signal transducer and activator of transcription 6/transcription factor EC/IL-4 receptor alpha positive feedback loop in M2 macrophages. J Allergy Clin Immunol 140(6): 1550-1561 e1558. 10.1016/j.jaci.2017.01.024

[5] Guo YC, Zhang M, Wang FX, et al. (2017) Macrophages Regulate Unilateral Ureteral Obstruction-Induced Renal Lymphangiogenesis through C-C Motif Chemokine Receptor 2-Dependent Phosphatidylinositol 3-Kinase-AKT-Mechanistic Target of Rapamycin Signaling and Hypoxia-Inducible Factor-1alpha/Vascular Endothelial Growth Factor-C Expression. Am J Pathol 187(8): 1736-1749. 10.1016/j.ajpath.2017.04.007

[6] Takiishi T, Cook DP, Korf H, et al. (2017) Reversal of Diabetes in NOD Mice by Clinical-Grade Proinsulin and IL-10-Secreting Lactococcus lactis in Combination With Low-Dose Anti-CD3 Depends on the Induction of Foxp3-Positive T Cells. Diabetes 66(2): 448-459. 10.2337/db15-1625

[7] Obata Y, Furusawa Y, Endo TA, et al. (2014) The epigenetic regulator Uhrf1 facilitates the proliferation and maturation of colonic regulatory T cells. Nature immunology 15(6): 571-579. 10.1038/ni.2886

[8] Yao Y, Wang Y, Zhang Z, et al. (2016) Chop Deficiency Protects Mice Against Bleomycin-induced Pulmonary Fibrosis by Attenuating M2 Macrophage Production. Mol Ther 24(5): 915-925. 10.1038/mt.2016.36

[9] Berg DJ, Zhang J, Weinstock JV, et al. (2002) Rapid development of colitis in NSAID-treated IL-10-deficient mice. Gastroenterology 123(5): 1527-1542

ESM Table 1 Primer sequences

Gene	Forward (5'-3')	Reverse (5'-3')
Ctla4	GCTTCCTAGATTACCCCTTCC	CGGGCATGGTTCTGGATCA
Pdcd1	ACCCTGGTCATTCACTTGGG	CATTTGCTCCCTCTGACACTG
Tgfb1	CTCCCGTGGCTTCTAGTGC	GCCTTAGTTTGGACAGGATG
Il10	GCTCTTACTGACTGGCATGAG	CGCAGCTCTAGGAGCATGTG
Il12a	CTGTGCCTTGGTAGCATCTAG	GCAGAGTCTCGCCATTATGAC
Ebi3	CTTACAGGCTCGGTGTGGC	GTGACATTTAGCATGTAGGGA
Actb	GGCTGTATTCCCCTCCATCG	CCAGTTGGTAACAATGCCAT

ESM Table 2 Clinical characteristics of the participants

	Control participants	Participants with T1D
Number	34	30
Age (years)	29.47±2.190	19.29±3.118
Sex (M/F)	11/23	18/12
HbA1c (mmol/mol)	37.57±3.176	83.29±12.99
HbA1c (%)	5.586±0.2907	9.759±1.184
FPG (mmol/l)	5.916±0.1576	8.410±1.158
2HG (mmol/l)	6.114±0.5822	25.44±1.637
FPI (pmol/l)	68.92±9.16	15.33±7.21
2HI (pmol/l)	258.63±42.66	23.06±14.69
Diabetes duration (months)	NA	6.4±2.9
Number of subjects with IAA^+	NA	19
Number of subjects with $GADA65^+$	NA	17
Number of subjects with $IAA^+GADA65^+$	NA	6

HbA1c, glycosylated hemoglobin; FPG, fasting plasma glucose; 2HG, 2-hour postprandial blood glucose; FPI, fasting plasma insulin; 2HI, 2-hour postprandial blood insulin.

ESM Figures

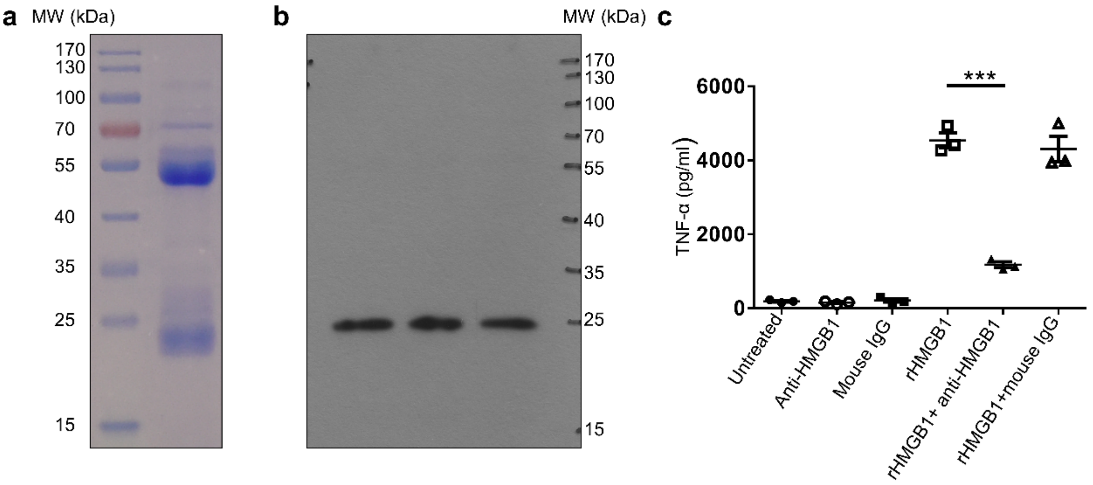

ESM Fig. 1 HMGB1-neutralising antibody was produced from peritoneal dropsy. (**a**) Coomassie staining of the anti-HMGB1 antibody purified through HiTrap Protein A HP. (**b**) Western blot analysis of anti-HMGB1 antibody purified through HiTrap Protein A HP. (**c**) RAW264.7 cells were used to test the neutralising effect of the antibody. rHMGB1-stimulated RAW264.7 cells secreted high levels of TNF-α, whereas blockade of HMGB1 by the neutralising antibody significantly inhibited TNF-α secretion. All in vitro studies were conducted 3 times. Values are expressed as mean ± SEM. Statistical difference was analysed by unpaired Student's *t* test; ****p*<0.001. MW, molecular weight

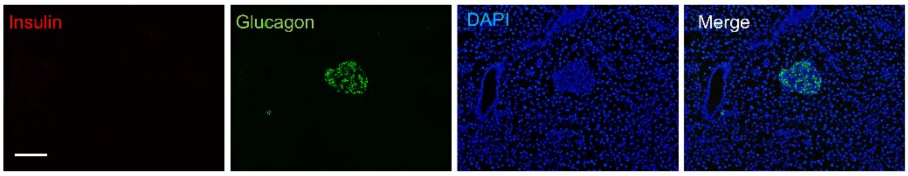

ESM Fig. 2 No functional beta cells were found in recipient pancreatic tissues of NOD females 4 weeks after they got diabetes. The figure is representative of 3 mice studied. Scale bar, 100μm. Original magnification ×200

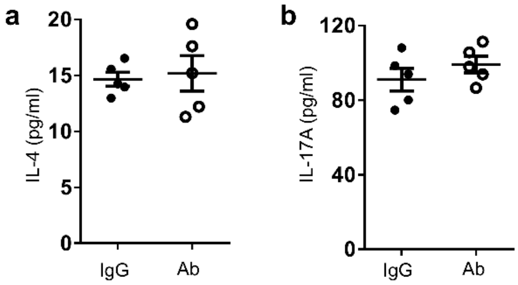

ESM Fig. 3 Cytokine production by PLN mononuclear cells was detected after anti-CD3 mAb stimulation *in vitro*. n=5 in each study group. Values are expressed as mean ± SEM. Statistical difference was analysed by unpaired Student's *t* test

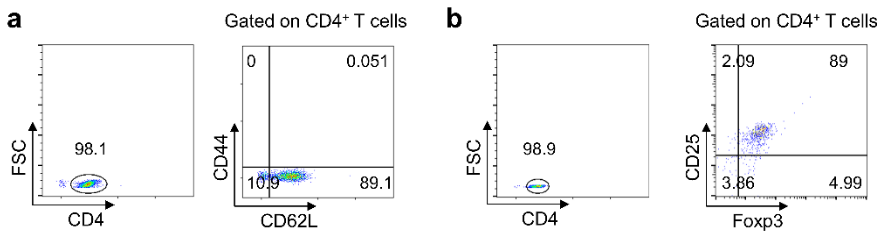

ESM Fig. 4 The purity of isolated naïve CD4$^+$ T cells (**a**) and CD4$^+$CD25$^+$ Tregs (**b**) from the spleen of NOD mice by MACS

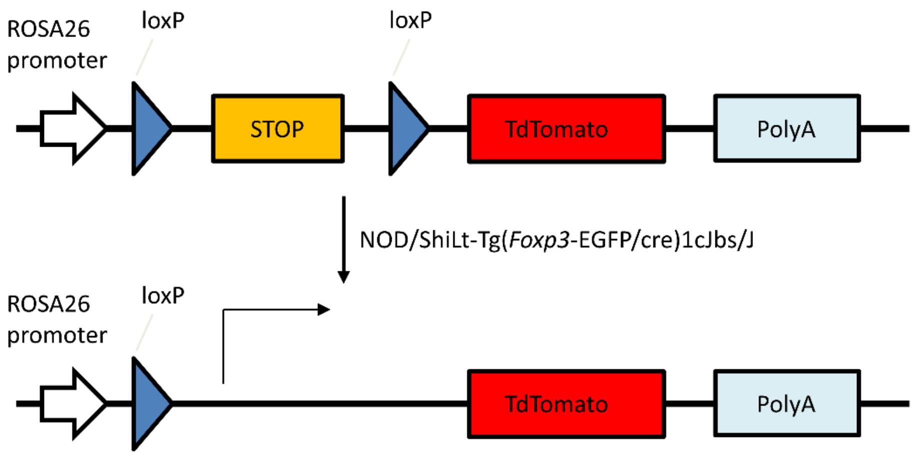

ESM Fig. 5 The transgenic reporter mice were generated using the indicated strategy. There is a Cre reporter allele that has a *loxP*-flanked STOP cassette preventing transcription of a CAG promoter-driven red fluorescent protein variant (tdTomato) inserted into the *Gt(ROSA)26Sor* locus in 007914-B6.Cg-*Gt(ROSA)26Sor*$^{tm14(CAG\text{-}tdTomato)Hze}$/J mice. We across the 008694-NOD/ShiLt-Tg(*Foxp3*-EGFP/cre)1cJbs/J mice with the 007914-B6.Cg-*Gt(ROSA)26Sor*$^{tm14(CAG\text{-}tdTomato)Hze}$/J mice. In the resultant mice, *Foxp3*-GFP-Cre^{+} Tregs excise the *loxP*-flanked STOP cassette to drive constitutive transcription of the gene encoding RFP from the R26 promoter, which permanently marks the FOXP3^{+} T cells and their progeny

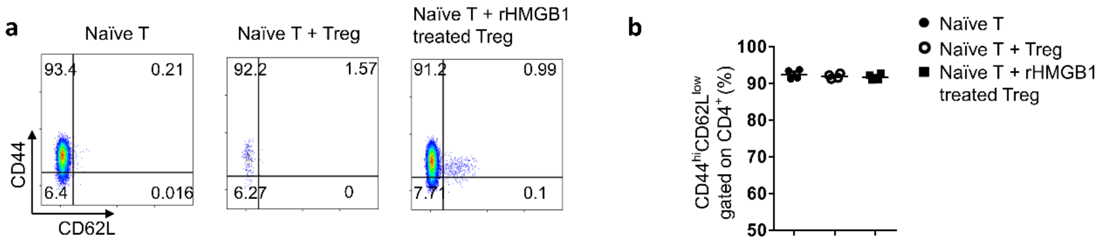

ESM Fig. 6 Representative FACS plots (**a**) and frequencies (**b**) of CD44 and CD62L expression by $CD4^{+}$ T cells in colon lamina propria 9 weeks after adoptive transfer. n=4/group. Values are expressed as mean ± SEM. Statistical difference was analysed by unpaired Student's *t* test

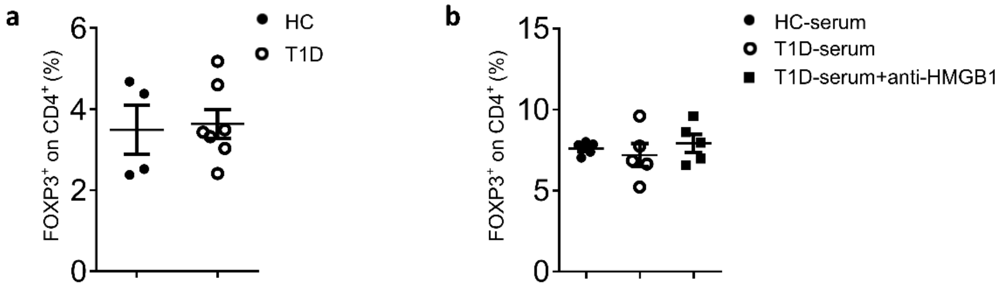

ESM Fig. 7 The frequencies of Tregs were shown in the indicated conditions. (**a**) Frequencies of FOXP3 expression by CD4$^+$ PBMC from diabetic (*n*=7) and control participants (*n*=4). (**b**) Percentages of FOXP3$^+$ cells within the CD4$^+$ PBMC during incubation of HC-PBMC with HC-serum or T1D-serum in the presence of HMGB1 neutralising antibody or not. *n*=5 per group. Values are expressed as mean ± SEM. Statistical difference was analysed by unpaired Student's *t* test. HC, healthy control; T1D, type 1 diabetes
